# Patient-specific implants made of 3D printed bioresorbable polymers at the point-of-care: material, technology, and scope of surgical application

**DOI:** 10.1186/s41205-024-00207-0

**Published:** 2024-04-19

**Authors:** Michaela Maintz, Céline Tourbier, Michael de Wild, Philippe C. Cattin, Michel Beyer, Daniel Seiler, Philipp Honigmann, Neha Sharma, Florian M. Thieringer

**Affiliations:** 1 Oral and Cranio-Maxillofacial Surgery, University Hospital Basel, Spitalstrasse 21, Basel, Switzerland; 2 Department of Biomedical Engineering, Medical Additive Manufacturing Research Group (Swiss MAM), University of Basel, Hegenheimermattweg 167C, Allschwil, Switzerland; 3 Institute for Medical Engineering and Medical Informatics IM², University of Applied Sciences and Arts Northwestern Switzerland FHNW, Hofackerstrasse 30, Muttenz, Switzerland; 4 Department of Biomedical Engineering, Center of Medical Image Analysis and Navigation (CIAN), University of Basel, Hegenheimermattweg 167C, Allschwil, Basel, Switzerland; 5 Department of Orthopaedic Surgery and Traumatology, Hand- and peripheral Nerve Surgery, Kantonsspital Baselland, Bruderholz| Liestal| Laufen, Switzerland; 6 Biomedical Engineering and Physics, Amsterdam UMC location University of Amsterdam, Meibergdreef 9, Amsterdam, The Netherlands

**Keywords:** Computer-aided design, Defect, Bone, Three-dimensional, 3D Printing, Polymers, Composites, Osteosynthesis, Regeneration, Lattice, Point-of-care, Hospital

## Abstract

**Background:**

Bioresorbable patient-specific additive-manufactured bone grafts, meshes, and plates are emerging as a promising alternative that can overcome the challenges associated with conventional off-the-shelf implants. The fabrication of patient-specific implants (PSIs) directly at the point-of-care (POC), such as hospitals, clinics, and surgical centers, allows for more flexible, faster, and more efficient processes, reducing the need for outsourcing to external manufacturers. We want to emphasize the potential advantages of producing bioresorbable polymer implants for cranio-maxillofacial surgery at the POC by highlighting its surgical applications, benefits, and limitations.

**Methods:**

This study describes the workflow of designing and fabricating degradable polymeric PSIs using three-dimensional (3D) printing technology. The cortical bone was segmented from the patient’s computed tomography data using Materialise Mimics software, and the PSIs were designed created using Geomagic Freeform and nTopology software. The implants were finally printed via Arburg Plastic Freeforming (APF) of medical-grade poly (L-lactide-co-D, L-lactide) with 30% β-tricalcium phosphate and evaluated for fit.

**Results:**

3D printed implants using APF technology showed surfaces with highly uniform and well-connected droplets with minimal gap formation between the printed paths. For the plates and meshes, a wall thickness down to 0.8 mm could be achieved. In this study, we successfully printed plates for osteosynthesis, implants for orbital floor fractures, meshes for alveolar bone regeneration, and bone scaffolds with interconnected channels.

**Conclusions:**

This study shows the feasibility of using 3D printing to create degradable polymeric PSIs seamlessly integrated into virtual surgical planning workflows. Implementing POC 3D printing of biodegradable PSI can potentially improve therapeutic outcomes, but regulatory compliance must be addressed.

## Background

Technological advancements and the desire for precision, flexibility, and targeted patient care have recently led to a significant leap in digitalization in patient-specific implants (PSIs) for oral and maxillofacial surgery [1]. PSIs are a popular alternative to conventional off-the-shelf implants and deliver improved clinical results, especially for patients with complex pathologies where standard treatment is not an option [2]. Moreover, PSIs for osteosynthesis have been shown to provide better stability and shorter operating time than standard implants [3]. A drawback of using standard resorbable plates is that the molding process for craniofacial reconstruction has been shown to reduce the strength and stiffness through accelerated hydrolysis after extended submersion in the heated bath [4]. Bone defects caused by trauma, infection, or tumor resection are usually treated with autologous bone transfer, currently considered the gold standard [5]. The drawbacks of such autografts include limited graft size, associated donor site morbidity, challenging accurate graft adaption [6], and the prolonged surgery time associated with postoperative complications [7, 8]. Recently, patient-specific bone tissue engineering and osteosynthesis methods have been further developed as promising alternatives to address the limitations of conventional approaches [6, 9, 10]. Combining preoperative planning with computer-aided design and PSIs can improve reconstructive accuracy [11] and save time during surgery [10, 12]. Furthermore, with three-dimensional (3D) printing of bioresorbable PSIs, the cumbersome and error-prone process of bending plates [13] and pre-shaping bone grafts [14] can be avoided. Several biomaterial options are available for the fabrication of biodegradable PSIs, the most popular being metals like magnesium [15], bioceramics such as tricalcium phosphate (TCP) [15], polymers like polylactic acid (PLA) [16], and various composites [17].

Metal biomaterials such as titanium are known to be highly durable and biocompatible and are widely used to fixate bone and support its regeneration. Nonetheless, additional removal surgery is required if the titanium implant is not intended for permanent placement. Additive manufacturing of biodegradable metals such as magnesium, iron, and zinc has shown promising results [18]. However, studies have indicated that enhancing the degradation properties and biological effects of resorbable metals in clinical applications often requires modifications such as alloying [14, 19–24].

Biodegradable bioceramics like β-TCP are another option. The ceramic biomaterials used in medicine are a homogenous group of usually biocompatible, non-toxic, hard, and brittle materials with high-temperature stability [25], such as alumina and zirconia. They are suitable for orthopedic implants and dental restorations due to their high strength and inertness [26]. Calcium phosphate bioceramics, such as hydroxyapatite and α-/β-TCP, along with bioactive glasses, are biodegradable [27]. However, in contrast to the bone, they exhibit high elastic moduli and usually high compressive but low tensile strength and are, therefore, not suitable for load-bearing applications [28].

The use of biodegradable polymers offers several advantages over traditional, non-biodegradable materials. Additional healthcare costs and complications can be avoided by eliminating the need for a second surgery for implant removal or surgical revisions due to implant-associated complications [29, 30]. This is particularly useful in applications such as suture material, scaffolds for tissue engineering, and osteosynthesis plates where the material is only needed for a limited healing time [31]. Synthetic polymers are a reliable source of innovative materials because of their ability to be adapted to a wide range of degradation rates, structural properties, and mechanical characteristics [32]. For example, the degradation kinetics of PLA, a commonly clinically used biodegradable polymer, can be tailored by adapting the molecular composition using L- or D-chirality. Diffusion of water within the polymer chains causes both surface and bulk degradation by a process called hydrolysis. This results in the formation of lactic acid or a combination of carbon dioxide and water. These degradation products can be metabolized within cells or expelled from the body through urine and breathing [33].

Due to their unique combination of mechanical, physical, and biological properties, composite materials have also become increasingly popular in medical applications. They are typically made of two or more specific materials combined to form a compound that exhibits superior properties supporting the regeneration [25]. In recent years, researchers have focused on developing composites that can be used for orthopedic, cranio-maxillofacial, and dental applications, particularly on biocompatible and biodegradable materials that can promote tissue regeneration [31]. Among the various types of composite materials studied for medical applications, PLA ceramic composites have shown promising results in promoting bone growth and regeneration [34, 35]. In an *in-vivo* critical bone defect rat study, PLA/β-TCP composites showed a superior ability to promote osteogenesis, particularly in the early stages of bone healing, as demonstrated by immunohistochemical analysis [36].

The design of individualized implants has become more user-friendly and efficient through the advancements of 3D imaging and visualization of computer-assisted design user interfaces. Consequently, since 3D printers are becoming increasingly precise, affordable, and easy to use, point-of-care (POC) 3D printing is a rapidly growing field in the medical industry. Producing patient-specific medical models at the POC, such as hospitals, clinics, and surgical centers, allows for a more flexible, faster, and more efficient process, reducing the need for outsourcing to external manufacturers [37]. 3D printing-based technologies additionally provide the capability to produce implants at the POC, a domain that is experiencing increasing attention [38–40]. Having expertise in designing 3D-printed implants in-house frees hospitals from the restrictions imposed by commercial availability [41] and can be advantageous or even necessary when there are time or delivery constraints. POC manufacturing facilitates face-to-face meetings among all stakeholders involved in the device’s planning and production, as well as generating prototypes in-house, and can accelerate the patient-specific implant (PSI) development process and offer innovative personalized treatment options [39, 42].

The creation of patient-specific resorbable 3D printed implants at the POC poses numerous challenges, such as designing, manufacturability, quality control, and regulatory compliance. Arburg Plastic Freeforming (APF) is a novel material jetting method developed by Arburg GmbH + Co KG (Lossburg, Germany), which can fabricate biodegradable polymeric implants. The APF method enables the use of medical-grade thermoplastic polymers and composites in the form of granules, typically used for injection molding. The advantage of APF for use at the POC is that the commercially available medical-grade material can be directly used for 3D printing. No additional thermoforming is required to create filament, minimizing further processing steps that could affect the part’s material properties. The open platform APF system facilitates the use of a wide range of materials such as PLA, polyether ether ketone (PEEK), poly vinyl alcohol (PVA), poly methyl methacrylate (PMMA) and composites. However, it requires the investigation of the effect of process parameters to optimize mechanical properties and print accuracy. This concept has been investigated for the processing of medical grade polymethyl methacrylate [43, 44] and PLA/hydroxyapatite scaffolds [45]. APF with bioresorbable PLA/β-TCP composites for additive manufacturing of PSI have not yet been investigated.

We present technical considerations and preliminary results on the 3D printing of implants using the APF technology for cranio-maxillofacial reconstruction. By exploring the scope of surgical applications, advantages, and limitations, we wish to highlight the potential benefits of producing bioresorbable polymer implants for cranio-maxillofacial surgery at the POC and to encourage further research and development in this field.

## Methods

### 3D model preparation

**Fig. 1:**
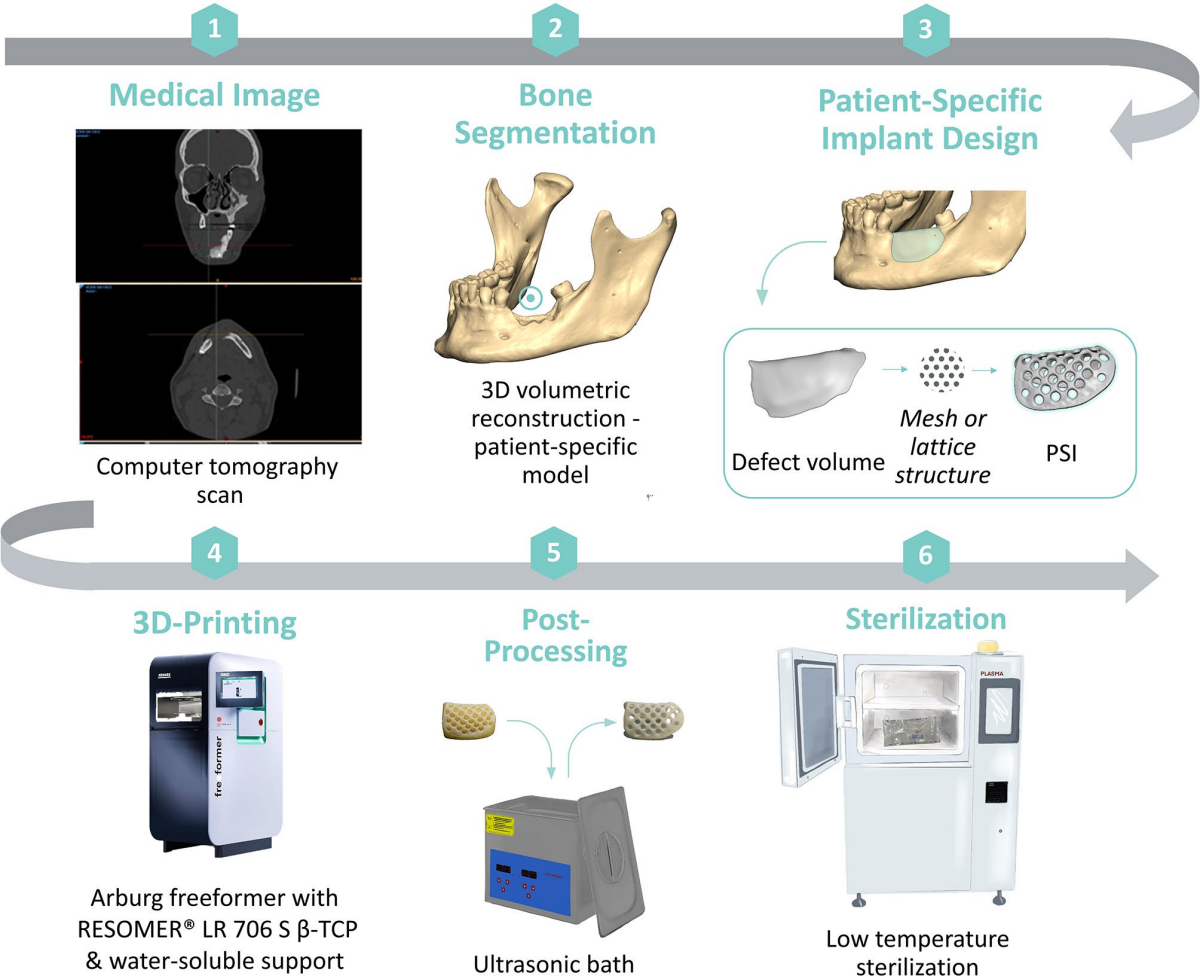
As an example, workflow of patient-specific design and fabrication with the mesh for mandibular alveolar bone regeneration. The process begins with the (1) retrieval of patient computed tomography (CT) imaging data and the (2) segmentation of relevant anatomical structures. Based on the segmented data, the (3) patient-specific implant (PSI) is designed. (4) The PSI is then three-dimensional (3D) printed from medical-grade raw material pellets. (5) The water-soluble support structures are removed in an ultrasonic bath. (6) Subsequently, the implant is packaged and then sterilized

The general workflow of PSI design and fabrication is depicted in Fig. 1. All 3D bone models were created using clinical computed tomography (CT) via Digital Imaging and Communications in Medicine (DICOM) raw data (Fig. 1 (1)). The cortical bone of the anonymized datasets was segmented using the Materialise Mimics software (Version 24.0, Materialise NV, Leuven, Belgium) by applying a threshold to create a mask of Hounsfield Unit (HU) gray values, which corresponded to cortical bone (Fig. 1 (2)). Non-relevant anatomical entities were excluded from the mask, and in the presence of metallic artifacts, minor modifications were conducted, such as smoothing and filling of the defects. After segmentation, the bone surface geometry was exported as a Standard Tessellation Language (STL) file. The PSI (Fig. 1 (3)) and the support structures were designed in Geomagic Freeform (Version 2021, 3D Systems, Rock Hill, South Carolina, United States).

The STL files of the implant designs and the support structures were imported to the Arburg Slicer software (Arburg GmbH + Co KG, Lossburg, Germany), where the two-dimensional (2D)-stacked slices of the implant and support geometries were prepared as an Arburg Freeformer Job (AFJ) file with the layer information.

### Arburg plastic freeforming

**Fig. 2:**
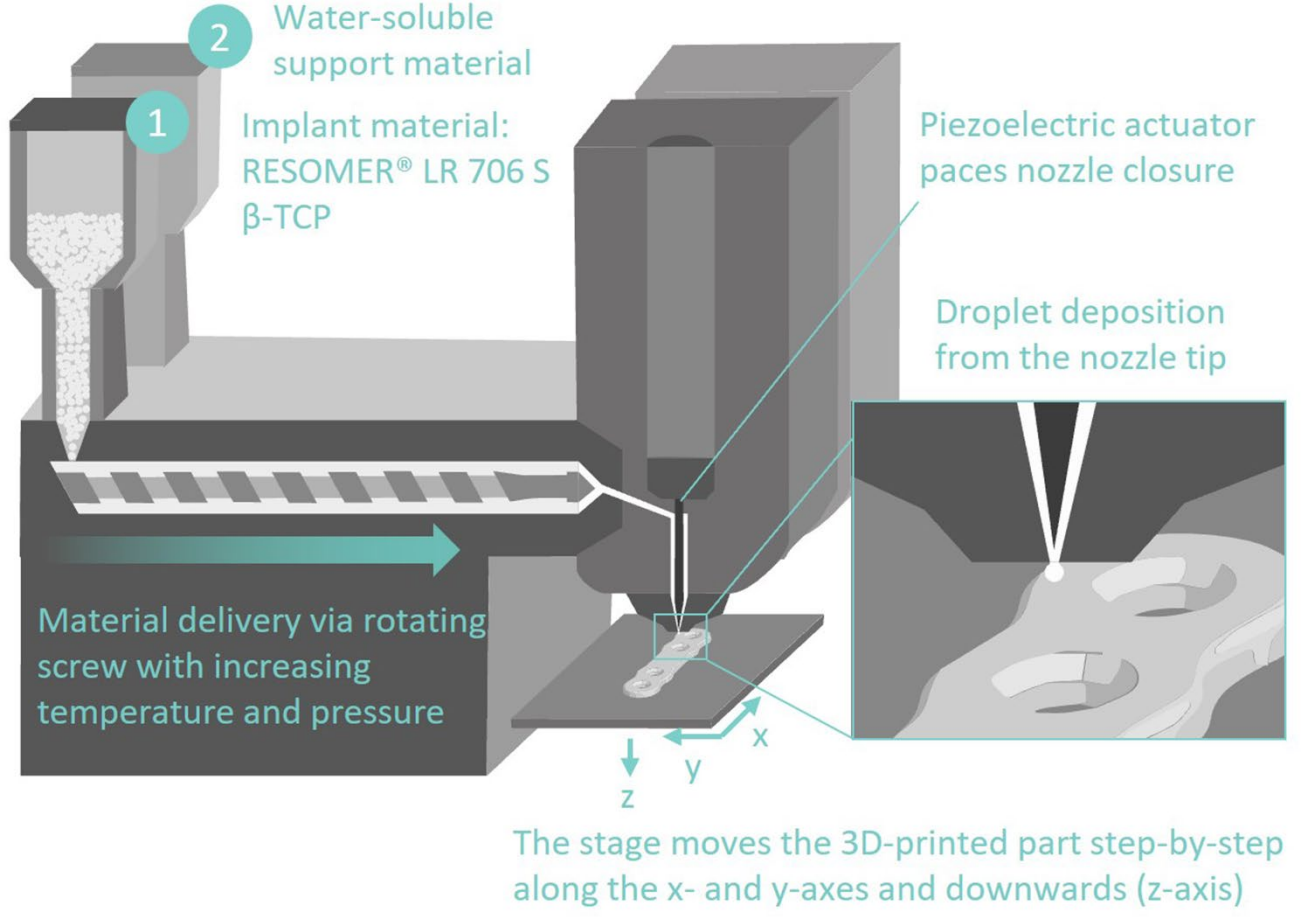
Schematic setup of the Arburg freeformer 200-3x, adapted from arburg.com, depicting the manufacturing process of a plate for fracture stabilization. The nozzle of discharge unit 1 deposits the implant material and the second nozzle of discharge unit 2 is used for the deposition of the water-soluble support material

All samples were 3D printed with the Arburg freeformer 200-3X (Arburg GmbH + Co KG, Lossburg, Germany, Fig. 1 (4)). As can be seen in Fig. 2, the APF process involves melting granulated polymers in different heating zones, applying them through a nozzle drop by drop, and building the desired part by lowering the stage along the z-axis after each layer deposition. The droplet diameter was ca. 200 μm (volume of approx. 4 × 10^6^ µm^3^). The entire process was conducted with a build chamber temperature of 85 ± 5 °C. The performance and technical specifications of the Arburg freeformer are listed in Table 1.

**Table 1:** Technical specifications of the Arburg freeformer 200-3x [47]

Parameter	Technical specifications
Usable build chamber space, 1-component (w, d, h)	max. 189 × 134 × 230 mm^3^
Usable build chamber space, 2-component (w, d, h)	max. 154 × 134 × 230 mm^3^
Layer thickness	0.2 mm
Wall thickness	0.6 mm
Absolute part precision (x and y)	± 0.1 mm
Discharge units	2
Nozzle diameter	0.2 mm
Discharge rate	2–14 max. cm³/h
Material pressure	1–800 bar
Material processing temperature	max. 350 °C
Build chamber temperature	max. 120 °C
Slicing software compatibility	Arburg freeformer software with integrated data processing of 3D geometries in STL format

For the fabrication of the bioresorbable PSI, RESOMER® LR 706 S β-TCP (Evonik Industries AG, Essen, Germany) granules were used. This biodegradable material is a composite containing 70:30 poly (L-lactide-co-D, L-lactide) with an additive of 30% β-tricalcium phosphate (PLDLLA/β-TCP). It offers a degradation time of under two years and is radiolucent [46]. Armat11 (Arburg GmbH + Co KG), a water-soluble polymer, was used for the support structures.

After the printing job, the stage is cooled to room temperature, and the PSI is detached. The 3D-printed support structures were removed by placing the 3D-printed parts in an ultrasonic bath filled with tap water for up to one hour (Fig. 1 (5)). The PSIs were then dried at room temperature, and if the implant required screws for fixation, the screw holes were manually drilled to ensure proper fit.

### Fabrication assessment

The layer pattern of the 3D-printed bioresorbable plate (length 26 mm, thickness 1.4 mm) was analyzed using (SEM) (TM3030Plus, Hitachi, Tokyo, Japan). The fit of the PSIs was visually assessed on a 3D printed PLA model of the patient’s bone (Replicator+, MakerBot, Brooklyn, USA) by manually checking its perfect placement (see Figs. 4b, 5a, 6a and 7c).

## Results

**Fig. 3:**
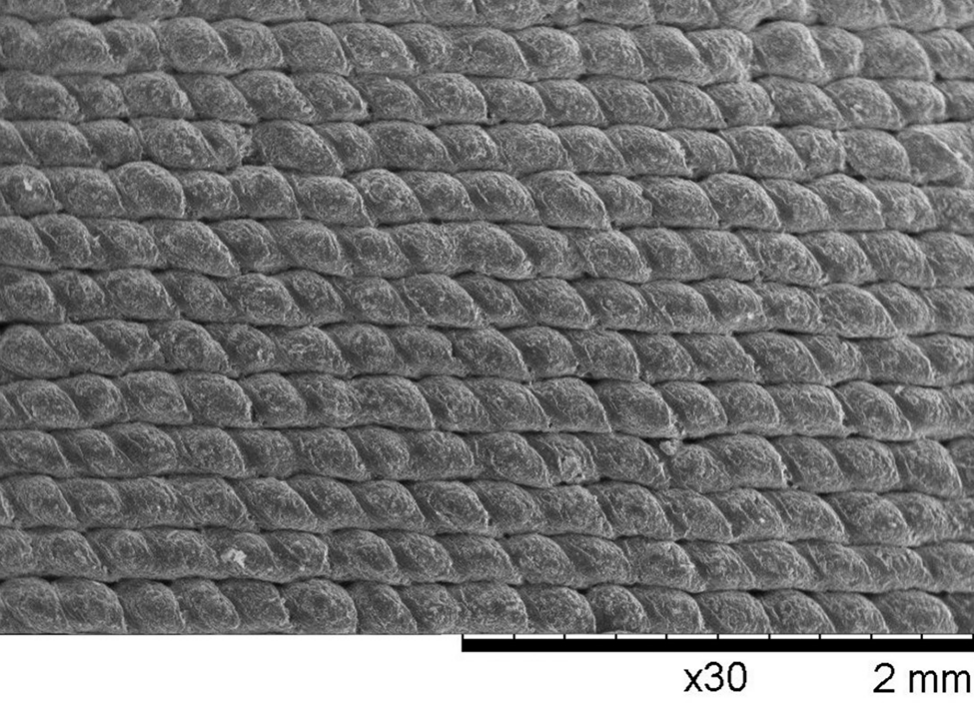
Scanning electron micrography (SEM) image of the surface structure of a three-dimensional (3D) printed patient-specific implant (PSI) showing a dense pattern of individual deposited droplets

**Fig. 4:**
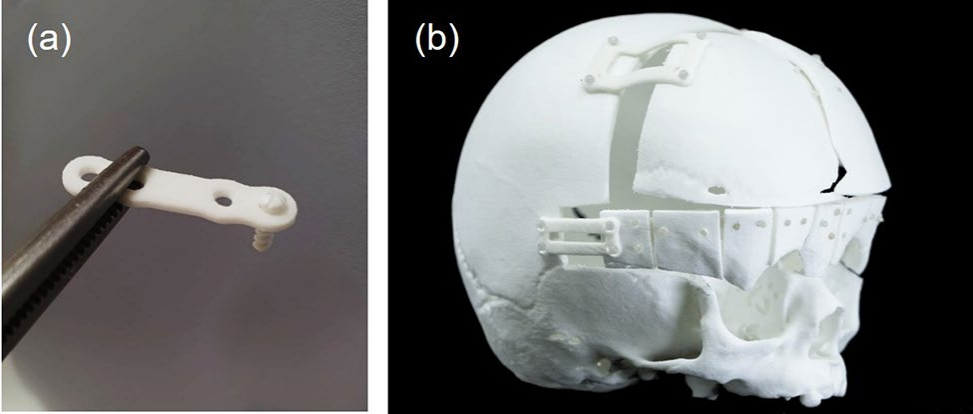
(**a**) Three-dimensional (3D)-printed bioresorbable plate (length 26 mm, thickness 1.4 mm) for osteosynthesis with commercially available bioresorbable standard screws (Osteotrans-MX, Teijin Medical Technologies, Osaka, Japan). (**b**) Patient-specific osteosynthesis plates for the fixation of advanced fronto-orbital bone segments

**Fig. 5:**
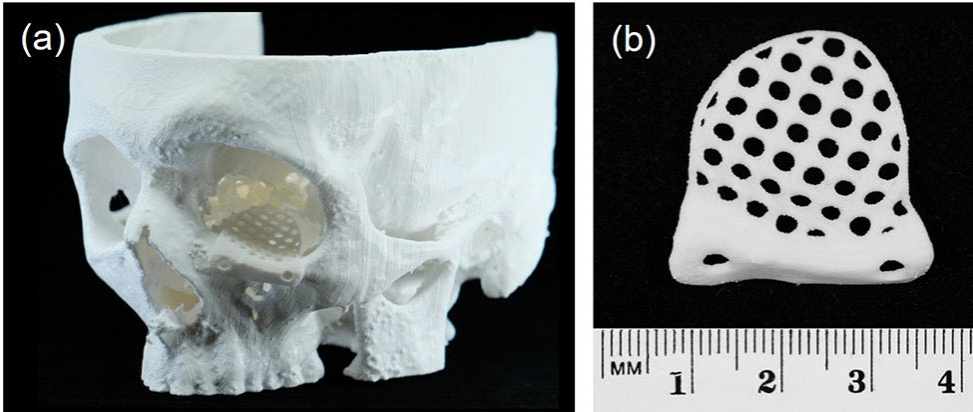
Low-profile (thickness 0.8 mm) patient-specific implant (PSI) for orbital bone fracture regeneration. (**a**) PSI mounted on the three-dimensional (3D) printed patient skull. (**b**) Close-up view of orbital mesh

**Fig. 6:**
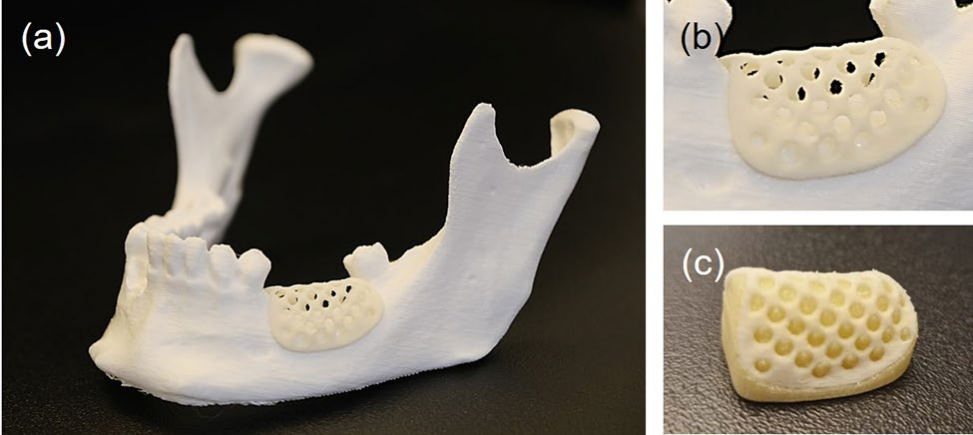
Three-dimensional (3D)-printed mesh (thickness 1.0 mm) for guided bone regeneration of the alveolar process. (**a**) Visual assessment of the implant fit on 3D-printed model. (**b**) Magnified view of the open-porous alveolar mesh. (**c**) Mesh with support structure before post-processing

**Fig. 7:**
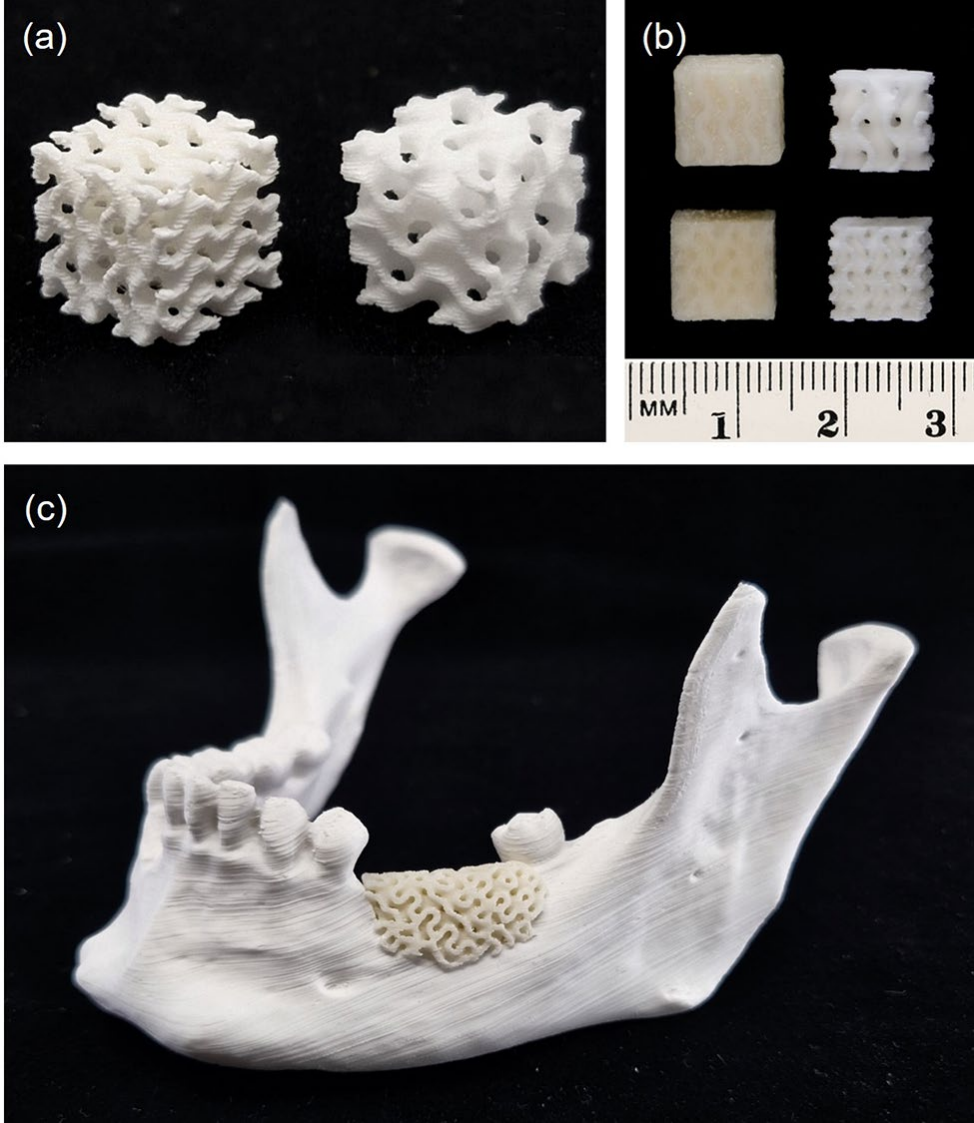
Bioresorbable composite scaffolds with a wall thickness of 1.0 mm and a channel width of approx. 0.5 mm (left) and 0.7 mm (right), additively manufactured using the Arburg Plastic Freeforming (APF) technology. (**a**) Isometric view of 10 × 10 × 10 mm^3^ scaffold with approx. 60% (left) and 70% (right) porosity. (**b**) Frontal view of scaffolds before (left) and after (right) removing the water-soluble support material. (**c**) Patient-specific implant (PSI) bone graft for alveolar bone defect, mounted on a three-dimensional (3D)-printed mandible

The SEM of the 3D-printed plate surface showed an extremely dense material with highly uniform and well-connected droplets with minimal gap formation between the printed paths (Fig. 3). Complex and curved structures were fabricated successfully within 5–60 min, and the water-soluble support material was dissolved entirely in an ultrasonic bath within one hour. In this preliminary investigation using the technology, the following parts were successfully 3D printed:

I. Plates for osteosynthesis (Fig. 4a and b).
II. Implants for orbital floor fractures (Fig. 5a and b).
III. Meshes for alveolar bone regeneration (Fig. 6a-c).
IV. Bone scaffolds with interconnected channels (Fig. 7a-c).

For the plates and meshes, a wall thickness down to 0.8 mm could be reached with good print quality. The fabrication of undersized screw holes and manual drilling of screw holes facilitated a perfect fit between the implant and the screws. The fit test on the adjacent bone models yielded minimal gap formation and no unwanted friction between the parts. These results indicate that it is possible to achieve geometrically accurate PSIs using APF technology.

## Discussion

3D printing has revolutionized how we approach medical treatments and has brought a new era of personalized medicine [48, 49]. The use of bioresorbable polymers and composites in 3D printing has further expanded the scope of application of this technology, with the overall goal to manufacture implants that reliably guide bone growth, provide suitable mechanical stability, and safely degrade within the body over time. Biodegradable fixation plates for maxillofacial trauma have demonstrated similar efficacy to titanium plates but with lower symptomatic removal rates, making them a viable alternative [29]. The biodegradable material should provide sufficient biomechanical stability while promoting bone growth and regeneration until the bone defect is healed. In this article, we present PSI designs for fronto-orbital bone advancement, meshes to treat alveolar defects of the mandibular bone, plates for orbital fractures, and scaffolds with interconnected micro-architectures.

Through tailor-made implant solutions created by a digital planning process, the bone-implant interface is improved through an optimal anatomical fit and suitable primary stability, which is especially relevant for treating cranio-maxillofacial deformities [50]. For example, patients with large orbital defects requiring surgical treatment with a titanium mesh stock implant are at risk of implant malpositioning [51]. Preformed titanium plates can give good outcomes in single-wall fractures but may not be sufficient to cover the defect in more extensive fractures involving multiple walls. In these cases, primary treatment with a PSI should be considered to reduce the risk of long-term sequelae due to inadequate reconstruction [52]. For orbital floor repair, a study by Goodson et al. [53] found that 83% of the participants perceived improvements in surgical duration, 84% in precision, and 69% in ease of placement when using 3D-printed titanium implants. Alloplastic materials such as titanium are persistent in foreign bodies and can induce infection, growth distortions in pediatric applications, persistent diplopia, and plate migration [54, 55]. Therefore, the use of bioresorbable implants for orbital reconstruction is gaining increased popularity. Clinical research has shown that conventional bioresorbable PLA implants are considered safe and effective for treating orbital fractures [56–58]. However, bioresorbable patient-specific orbital implants have not yet been thoroughly researched.

### 3D printing

The APF process was straightforward and could be easily linked to the standard virtual surgical planning procedure. APF ensured the fabrication of geometrically accurate parts with a layer height of 0.2 mm. The second nozzle facilitates using a water-soluble support material like PVA, which could be rapidly dissolved in water. The support structures enable the reproducible generation of concave overhangs and highly complex 3D structures, such as lattice structures for bone grafts or patient-specific plates for osteosynthesis to fit the bone surface optimally. Using APF offers the advantage of bypassing the requirement for pre-producing filaments from material powder or pellets, thereby reducing the thermal influence on material stability and degradation by changing molecular mass, chain length, or crystallinity. Moreover, APF technology allows for more precise control of drop deposition compared to fused filament fabrication using an extrusion screw [45].

However, the implementation of APF to produce bioresorbable PSIs comes with certain limitations. The open system of APF technology allows the adaptation of numerous process parameters, which is an advantage for the manufacturability of a large variety of thermoplastic materials. However, if a new material is used, the optimal parameter set for each material must first be systematically determined [59]. To improve the mechanical performance of the 3D printed part, the bond between the droplets must be strengthened, for example, through temperature adaptation or overfilling, which could cause geometric inaccuracies. Therefore, a compromise between internal density and surface quality must be made [44]. It was recognized that thermal effects influence the part geometry during the 3D printing process. In the standby mode of the Arburg freeformer, the material was stored in a molten state at temperatures of approx. 100 °C within the material transport unit. Therefore, before each print job, the thermally pretreated material was flushed for several minutes and discarded before the 3D printing process. Flushing was performed after idle phases of two hours or more, resulting in several grams of material expenditure. To achieve the best possible surface quality and mechanical strength next to the initial 3D print parameter optimization, re-scaling is often necessary to compensate for shrinkage when parts of different geometries are produced.

### Point-of-care manufacturing

Currently, commercial implants are mass-produced with only a limited available range of sizes and shapes. The hospital chooses the type of implants and orders the respective amount. After the implants arrive at the hospital, they are stored at a central warehouse or directly near the operating theatres. This process is not sustainable due to the high amount of unused products and the low demand for specific sizes [24]. Additionally, today, only a few manufacturing sites that produce PSIs are located near hospitals [40]. For the POC manufacturing of PSIs, specialized equipment, software, and trained personnel are needed, which can be expensive and are usually not readily available in healthcare facilities. Therefore, most processes are outsourced to external suppliers [40]. The production of PSIs at the POC would alleviate the local inventory burden and reduce dependence on global supply chain networks [60]. For simple reconstructions for patient-specific orbital fractures investigated by Korn et al. [61] the in-house training of engineers for PSI design has been shown to simplify the communication process with the surgeons and, therefore, improve the planning workflow and effectiveness, leading to faster and more efficient PSI production. The mean duration of PSI manufacturing was reduced from approx. 14 days (engineer with no in-house training) to approx. ten days (with in-house training). Consequently, the synergetic effect of the engineer’s involvement in the preoperative planning, clinical environment, close contact with medical staff, and the possibility of visiting the operating room to investigate the surgical process and implantation could increase their knowledge of the process, leading to more effective PSI designs.

However, unlike the manufacturing of anatomical models, the in-house manufacturing of implants remains challenging since many hospitals lack financial resources and regulatory guidance to implement the designing and manufacturing processes [40]. Studies have shown that in-house printing of anatomical models and surgical guides shortens the duration between surgical planning and surgery by avoiding the time-consuming intraoperative plate adaptation [62], transportation, and queue time of external suppliers [63]. In the literature review of Murtezani et al. [64] 96.89% of the studies involving 3D printing in cranio-maxillofacial surgery reported a satisfying or even better outcome using 3D printing. Considering the reduced surgery time, the hospitals seemed to save expenses compared to conventional approaches. Seeing the sinking prices of 3D printing technology and the increasing availability of affordable surgical planning tools, we estimate that the financial burden will soon be relatively low compared to the benefit of implementing POC 3D printing in hospitals to produce anatomical models, surgical guides, and even individualized implants.

Additional investigation is required to comprehensively evaluate the fit accuracy, structural integrity and biomechanical properties of the PSIs. Furthermore, future plans should include an assessment of in vitro and in vivo degradation as well as resorption behavior of the material. Nonetheless, literature suggests that the PLDLLA/β-TCP composite is suitable for bone tissue engineering applications. For example, the PLDLLA/β-TCP (90% and 10%wt) composite 3D printed scaffold by Lam et al. [35] has shown good in vitro biocompatibility and compressive strength of 3 MPa, similar to cancellous bone. Another study that has used 3D printed β-TCP scaffolds with PLDLLA infiltration has also demonstrated good biocompatibility with a cell survival rate of approximately 80% and compressive stress of 7.4 MPa [34].

### Outlook

With advancements in biomaterials research and digitalization, 3D printing can increasingly mimic the structure and function of natural bone, further enabling more personalized and effective bone regeneration. POC manufacturing could allow for higher flexibility and efficiency in complex bone reconstructions where standard treatments would fail. Nevertheless, when 3D printing PSIs at the POC, meeting national regulatory requirements, such as given by the Medical Device Regulation (MDR) [65] or the Food and Drug Administration (FDA) [66, 67], is crucial. Furthermore, *in silico* validation methods such as finite element analysis can be used to preoperatively assess the PSI’s effectiveness by incorporating the knowledge of mechanical properties, implant resorption, and bone growth mechanisms [68–70]. With optimization methods, the implant design could be further personalized to improve the surgical outcome [71].

We expect that design and manufacturing processes will continue to be streamlined through advances in artificial intelligence and process automation, enabling an increasing number of clinics to gain access to 3D printing means for bioresorbable PSIs [62].

## Conclusions

As 3D printing technology continues to advance and gain use in hospitals, the appeal of in-house production of PSIs is growing. In this study, we demonstrate the feasibility of creating PSIs with a good fit. The production process can be seamlessly linked to the established virtual surgical planning workflow. Despite being in its early stages, the integration of biodegradable 3D printing in PSI creation shows strong potential for improving the therapeutic outcome. Further innovation in this field is expected as 3D printing technologies and virtual surgical planning software are being further developed. It is crucial to consider regulatory requirements during the research and development phase to ensure compliance. However, since many hospitals currently lack the regulatory guidance to implement these processes, addressing challenges related to regulatory compliance and standardization becomes imperative. With this article, we hope to inspire continued exploration and collaboration among researchers, clinicians, and regulatory bodies to pave the way for the responsible and effective integration of biodegradable 3D printing in hospitals.

## Data Availability

The datasets used in the study are available from the corresponding author on reasonable request.

## Abbreviations

2D: Two-Dimensional
3D: Three-Dimensional
APF: Arburg Plastic Freeforming
AFJ: Arburg Freeformer Job
CT: Computed Tomography
DICOM: Digital Imaging and Communications in Medicine
FDA: Food and Drug Administration
HU: Hounsfield Unit
MDR: Medical Device Regulation
PEEK: Polyether Ether Ketone
PLA: Polylactic Acid
PLDLLA: Poly (L-lactide-co-D, L-lactide)
PMMA: Poly Methyl Methacrylate
PVA: Poly Vinyl Alcohol
POC: Point-Of-Care
PSI: Patient-Specific Implant
PSIs: Patient-Specific Implants
SEM: Scanning Electron Micrography
STL: Standard Tessellation Language
TCP: Tricalcium Phosphate

## References

[1] Huang MF, Alfi D, Alfi J, Huang AT (2019). The Use of patient-specific implants in oral and maxillofacial surgery. Oral Maxillofacial Surg Clin.

[2] Wong KC (2016). 3D-printed patient-specific applications in orthopedics. Orthop Res Rev.

[3] Taalab DA, Shehab AF, Atef M, Shehab MF (2023). Comparative study between patient specific titanium plates versus conventional miniplates for treatment of mandibular fractures: randomized clinical trial. J Cranio-Maxillofacial Surg.

[4] Ballard TNS (2010). Absorbable plate strength loss during molding. J Craniofac Surg.

[5] Pape HC, Evans A, Kobbe P. Autologous bone graft: Properties and techniques. J Orthop Trauma. 2010;24. 10.1097/BOT.0b013e3181cec4a1. 10.1097/BOT.0b013e3181cec4a1 20182233

[6] Kobbe P, Laubach M, Hutmacher DW, Alabdulrahman H, Sellei RM, Hildebrand F (2020). Convergence of scaffold-guided bone regeneration and RIA bone grafting for the treatment of a critical-sized bone defect of the femoral shaft. Eur J Med Res.

[7] Irawati N (2023). Effect of operative time on complications associated with free flap reconstruction of the head and neck. Clin Otolaryngol.

[8] Hardy KL (2014). The impact of Operative Time on complications after plastic surgery: a Multivariate Regression Analysis of 1753 cases. Aesthetic Surg J.

[9] Han HH et al. Reconstruction of Complex Maxillary Defects Using Patient-specific 3D-printed Biodegradable Scaffolds, *Plast Reconstr Surg Glob Open*, vol. 6, no. 11, p. e1975, 2018, 10.1097/gox.0000000000001975. 10.1097/GOX.0000000000001975 PMC6414092 30881789

[10] Essig H (2017). Patient-specific biodegradable implant in pediatric craniofacial surgery. J Cranio-Maxillofacial Surg.

[11] U VN, Mehrotra D, Howlader D, Singh PK, Gupta S (2019). Patient specific three-Dimensional Implant for Reconstruction of Complex Mandibular defect. J Craniofac Surg.

[12] Sánchez-Jáuregui E, Baranda- Manterola E, Ranz- Colio Á, Bueno de Á, Vicente, Acero J, Sanz (2022). Custom made cutting guides and osteosynthesis plates versus CAD/CAM occlusal splints in positioning and fixation of the maxilla in orthognathic surgery: a prospective randomized study. J Cranio-Maxillofacial Surg.

[13] Tatum SA (2012). Retrospective review of resorbable plate fixation in pediatric craniofacial surgery: long-term outcome. Arch Facial Plast Surg.

[14] Zhang X, Li X-W, Li J-G, Sun X-D (2014). Preparation and mechanical property of a novel 3D porous magnesium scaffold for bone tissue engineering. Mater Sci Engineering: C.

[15] Prakasam M, Locs J, Salma-Ancane K, Loca D, Largeteau A, Berzina-Cimdina L. Biodegradable materials and metallic Implants-A review. J Funct Biomater. 2017;8(4). 10.3390/jfb8040044. 10.3390/jfb8040044 PMC5748551 28954399

[16] Liu S, Qin S, He M, Zhou D, Qin Q, Wang H (2020). Current applications of poly(lactic acid) composites in tissue engineering and drug delivery. Compos Part B: Eng.

[17] Modrák M, Trebuňová M, Balogová AF, Hudák R, Živčák J (2023). Biodegradable materials for tissue Engineering: Development, classification and current applications. J Funct Biomaterials.

[18] Xia D, Yang F, Zheng Y, Liu Y, Zhou Y (2021). Research status of biodegradable metals designed for oral and maxillofacial applications: a review. Bioactive Mater.

[19] Peng Q, Huang Y, Zhou L, Hort N, Kainer KU. Preparation and properties of high purity Mg–Y biomaterials, *Biomaterials*, vol. 31, no. 3, pp. 398–403, 2010, 10.1016/j.biomaterials.2009.09.065. 10.1016/j.biomaterials.2009.09.065 19800117

[20] Zberg B, Uggowitzer PJ, Löffler JF (2009). MgZnCa glasses without clinically observable hydrogen evolution for biodegradable implants. Nat Mater.

[21] Kraus T (2014). Biodegradable Fe-based alloys for use in osteosynthesis: outcome of an in vivo study after 52weeks. Acta Biomater.

[22] Katarivas Levy G, Goldman J, Aghion E. The Prospects of Zinc as a Structural Material for Biodegradable Implants—A Review Paper, *Metals*, vol. 7, no. 10, p. 402, 2017, 10.3390/met7100402.

[23] Zhang J (2021). Biodegradable metals for bone defect repair: a systematic review and meta-analysis based on animal studies. Bioactive Mater.

[24] Chua MCH, Chui C-K (2016). Optimization of patient-specific design of medical implants for manufacturing. Procedia CIRP.

[25] Holzapfel BM (2013). How smart do biomaterials need to be? A translational science and clinical point of view. Adv Drug Deliv Rev.

[26] Giordano Ii R (2022). Ceramics overview. Br Dent J.

[27] Hing KA (2005). Bioceramic bone graft substitutes: influence of Porosity and Chemistry. Int J Appl Ceram Technol.

[28] Eliaz N, Metoki N. Calcium Phosphate Bioceramics: A Review of Their History, Structure, Properties, Coating Technologies and Biomedical Applications, *Materials*, vol. 10, no. 4, 2017, 10.3390/ma10040334. 10.3390/ma10040334 PMC5506916 28772697

[29] Gareb B, van Bakelen NB, Dijkstra PU, Vissink A, Bos RRM, van Minnen B (2020). Biodegradable versus titanium osteosynthesis in maxillofacial traumatology: a systematic review with meta-analysis and trial sequential analysis. Int J Oral Maxillofac Surg.

[30] Gareb B, Van Bakelen NB, Vissink A, Bos RRM, Van Minnen B. Titanium or Biodegradable Osteosynthesis in Maxillofacial Surgery? In Vitro and In Vivo Performances, *Polymers*, vol. 14, no. 14, p. 2782, 2022, 10.3390/polym14142782. 10.3390/polym14142782 PMC9316877 35890557

[31] Li C (2020). Design of biodegradable, implantable devices towards clinical translation. Nat Reviews Mater.

[32] Liu X, Ma PX. Polymeric scaffolds for bone tissue engineering, *Ann Biomed Eng*, vol. 32, no. 3, pp. 477– 86, 2004, 10.1023/b:abme.0000017544.36001.8e. 10.1023/b:abme.0000017544.36001.8e 15095822

[33] da Silva D (2018). Biocompatibility, biodegradation and excretion of polylactic acid (PLA) in medical implants and theranostic systems. Chem Eng J.

[34] Cornelsen M (2017). Mechanical and biological effects of infiltration with biopolymers on 3D printed tricalciumphosphate scaffolds. Dent Mater J.

[35] Lam C, Olkowski R, Swieszkowski W, Tan K, Gibson I, Hutmacher D. Composite PLDLLA/TCP scaffolds for bone engineering: mechanical and in vitro evaluations, in *13th International Conference on Biomedical Engineering*, 2009: Springer, pp. 1480–1483.

[36] Toda E et al. Feasibility of Application of the Newly Developed Nano-Biomaterial, β-TCP/PDLLA, in Maxillofacial Reconstructive Surgery: A Pilot Rat Study, *Nanomaterials*, vol. 11, no. 2, p. 303, 2021, 10.3390/nano11020303. 10.3390/nano11020303 PMC7912080 33503931

[37] Ballard DH, Mills P, Duszak R, Weisman JA, Rybicki FJ, Woodard PK. Medical 3D Printing Cost-Savings in Orthopedic and Maxillofacial Surgery: Cost Analysis of Operating Room Time Saved with 3D Printed Anatomic Models and Surgical Guides, *Academic Radiology*, vol. 27, no. 8, pp. 1103–1113, 2020, 10.1016/j.acra.2019.08.011. 10.1016/j.acra.2019.08.011 PMC7078060 31542197

[38] Zhao H (2017). Printing@Clinic: from Medical models to organ implants. ACS Biomaterials Sci Eng.

[39] Willemsen K, Nizak R, Noordmans HJ, Castelein RM, Weinans H, Kruyt MC (2019). Challenges in the design and regulatory approval of 3D-printed surgical implants: a two-case series. Lancet Digit Health.

[40] Martelli N et al. Advantages and disadvantages of 3-dimensional printing in surgery: a systematic review, *Surgery*, vol. 159, no. 6, pp. 1485–1500, 2016. [Online]. Available: https://www.sciencedirect.com/science/article/pii/S0039606015010557?via%3Dihub. 10.1016/j.surg.2015.12.017 26832986

[41] Calvo-Haro JA (2021). Conceptual evolution of 3D printing in orthopedic surgery and traumatology: from do it yourself to point of care manufacturing. BMC Musculoskelet Disord.

[42] Willemsen K, Nizak R, Noordmans HJ, Castelein RM, Weinans H, Kruyt MC. Challenges in the design and regulatory approval of 3D-printed surgical implants: a two-case series. Lancet Digit Health, 1, 4, pp. e163-e171, 2019. 10.1016/S2589-7500(19)30067-6 33323186

[43] Hentschel L, Kynast F, Petersmann S, Holzer C, Gonzalez-Gutierrez J. Processing Conditions of a Medical Grade Poly(Methyl Methacrylate) with the Arburg Plastic Freeforming Additive Manufacturing Process, *Polymers*, vol. 12, no. 11, p. 2677, 2020, 10.3390/polym12112677. 10.3390/polym12112677 PMC7696228 33198390

[44] Hentschel L, et al. Parameter optimization of the ARBURG Plastic Freeforming process by means of a design of experiments Approach. Adv Eng Mater. 2022;2200279. 10.1002/adem.202200279.

[45] Bayart M et al. Pellet-Based Fused Filament Fabrication (FFF)-Derived Process for the Development of Polylactic Acid/Hydroxyapatite Scaffolds Dedicated to Bone Regeneration, *Materials*, vol. 15, no. 16, p. 5615, 2022, 10.3390/ma15165615. 10.3390/ma15165615 PMC9415795 36013752

[46] RESOMER®, BIORESORBABLE POLYMERS FOR MEDICAL DEVICES. EVONIK. https://healthcare.evonik.com/en/medical-devices/bioresorbable-polymers/standard-polymers (accessed 08 May, 2023).

[47] freeformer 200-3X. ARBURG. https://www.arburg.com/media/daten/publications/technical_data/additive_manufacturing/arburg_freeformer_200-3x_td_680838_en_gb.pdf (accessed 08 May, 2023).

[48] Vaz VM, Kumar L (2021). 3D Printing as a Promising Tool in Personalized Medicine. AAPS PharmSciTech.

[49] Prendergast ME, Burdick JA (2020). Recent advances in enabling technologies in 3D printing for precision medicine. Adv Mater.

[50] Alonso-Rodriguez E, Cebrián JL, Nieto MJ, Del Castillo JL, Hernández-Godoy J, Burgueño M (2015). Polyetheretherketone custom-made implants for craniofacial defects: report of 14 cases and review of the literature. J Craniomaxillofac Surg.

[51] Nikunen M, Rajantie H, Marttila E, Snäll J (2021). Implant malposition and revision surgery in primary orbital fracture reconstructions. J Cranio-Maxillofacial Surg.

[52] Schlittler F, Vig N, Burkhard JP, Lieger O, Michel C, Holmes S (2020). What are the limitations of the non-patient-specific implant in titanium reconstruction of the orbit?. Br J Oral Maxillofac Surg.

[53] Goodson AMC (2021). Printed titanium implants in UK craniomaxillofacial surgery. Part II: perceived performance (outcomes, logistics, and costs). Br J Oral Maxillofac Surg.

[54] Jordan DR, St. Onge P, Anderson RL, Patrinely JR, Nerad JA. Complications Associated with Alloplastic Implants used in Orbital Fracture Repair, *Ophthalmology*, vol. 99, no. 10, pp. 1600–1608, 1992, 10.1016/S0161-6420(92)31760-9. 10.1016/s0161-6420(92)31760-9 1454329

[55] Brown AE, Banks P (1993). Late extrusion of alloplastic orbital floor implants. Br J Oral Maxillofac Surg.

[56] Lieger O, Schaller B, Zix J, Kellner F, Iizuka T (2010). Repair of orbital floor fractures using bioresorbable poly-L/DL-lactide plates. Arch Facial Plast Surg.

[57] Young SM, Sundar G, Lim TC, Lang SS, Thomas G, Amrith S (2017). Use of bioresorbable implants for orbital fracture reconstruction. Br J Ophthalmol.

[58] Al-Sukhun J, Törnwall J, Lindqvist C, Kontio R (2006). Bioresorbable Poly-l/dl-Lactide (P[L/DL]LA 70/30) plates are Reliable for repairing large Inferior Orbital Wall Bony defects: a pilot study. J Oral Maxillofac Surg.

[59] Hirsch A, Hecker F, Moritzer E. Process parameter optimization to improve the mechanical properties of Arburg Plastic Freeformed components, in *2019 International Solid Freeform Fabrication Symposium*, 2019: University of Texas at Austin, 10.26153/tsw/17308.

[60] Teo AQA, Ng DQK, Peng L, O’NEILL GK (2021). Point-of-care 3D Printing: a feasibility study of using 3D Printing for Orthopaedic Trauma. Injury.

[61] Korn P, Jehn P, Nejati-Rad N, Winterboer J, Gellrich N-C, Spalthoff S (2022). Pitfalls of surgeon-engineer communication and the Effect of In-House engineer training during Digital Planning of patient-specific implants for Orbital Reconstruction. J Oral Maxillofac Surg.

[62] King BJ, Park EP, Christensen BJ, Danrad R. On-Site 3-Dimensional Printing and Preoperative Adaptation Decrease Operative Time for Mandibular Fracture Repair, *Journal of Oral and Maxillofacial Surgery*, vol. 76, no. 9, pp. 1950.e1-1950.e8, 2018, 10.1016/j.joms.2018.05.009. 10.1016/j.joms.2018.05.009 29859953

[63] Williams FC, Hammer DA, Wentland TR, Kim RY (2020). Immediate Teeth in Fibulas: planning and Digital Workflow with Point-of-care 3D Printing. J Oral Maxillofac Surg.

[64] Murtezani I, Sharma N, Thieringer FM (2022). Medical 3D printing with a focus on point-of-care in Cranio- and maxillofacial surgery. A systematic review of literature. Annals 3D Print Med.

[65] Regulation (EU). 2017/745 of the European Parliament and of the Council of 5 April 2017 on medical devices, amending Directive 2001/83/EC, Regulation (EC) 178/2002 and regulation (EC) 1223/2009 and repealing Council directives 90/385/EEC and 93/42/EEC (text with EEA relevance.), 2017.

[66] Di Prima M, Coburn J, Hwang D, Kelly J, Khairuzzaman A, Ricles L. Additively manufactured medical products– the FDA perspective, *3D Printing in Medicine*, vol. 2, no. 1, p. 1, 2016, 10.1186/s41205-016-0005-9. 10.1186/s41205-016-0005-9 PMC6027614 29974058

[67] 3D Printing Medical Devices at the Point of Care. Discussion Paper. U.S. Food and Drug Administration. https://www.fda.gov/media/154729/download (accessed 08 May, 2023).

[68] Vautrin A, Wesseling M, Wirix-Speetjens R, Gomez-Benito MJ (2021). Time-dependent in silico modelling of orthognathic surgery to support the design of biodegradable bone plates. J Mech Behav Biomed Mater.

[69] Chandra G, Pandey A (2021). Design approaches and challenges for biodegradable bone implants: a review. Expert Rev Med Devices.

[70] Ansoms P, Barzegari M, Sloten JV, Geris L (2023). Coupling biomechanical models of implants with biodegradation models: a case study for biodegradable mandibular bone fixation plates. J Mech Behav Biomed Mater.

[71] Mehboob H, Chang S-H (2015). Optimal design of a functionally graded biodegradable composite bone plate by using the Taguchi method and finite element analysis. Compos Struct.

[72] swissethics. Guidance document for the researchers for the conduct of basic research projects. https://swissethics.ch/en/news/2020/07/16/leitlinien-fuer-forschende-in-der-grundlagenforschung (accessed.

